# Aftershock: CEO Great Famine Experience and Stock Price Crash Risk

**DOI:** 10.3389/fpsyg.2021.816034

**Published:** 2021-12-20

**Authors:** Fang Cheng, Wenjuan Ruan, Guoliang Huang, Liangliang Zhang

**Affiliations:** ^1^School of Economics and Management, China University of Mining and Technology, Xuzhou, China; ^2^Xuhai College, China University of Mining and Technology, Xuzhou, China; ^3^Teesside University, Middlesbrough, United Kingdom

**Keywords:** stock price crash risk, traumatic experience, CEO, early-life, Great Famine

## Abstract

This study examines the effect of CEOs’ early-life traumatic experience on firm-specific stock price crash risk. Drawing on the idea of natural experiments, we take the Great Famine in China as an external traumatic event which cannot be selected or controlled by human. The analysis points out that compensation psychology and irrational defense psychology after the trauma of Great Famine are important factors that cause CEOs to hoard bad news. Based on a large sample of Chinese companies from 2007 to 2017, we find evidence that CEOs who experienced the Great Famine during early-life tend to hoard bad news, which result in higher stock price crash risk. The more severe and prolonged the Great Famine that the CEOs experienced, the greater the effect of this traumatic experience. CEOs decision-making power enhances the adverse effect of CEOs’ early-life traumatic experiences on crash risk. Findings of this study contributes to the literature by providing a new explanation for the stock price crash risk, which is of great significance for the sustained and healthy development of capital markets.

## Introduction

The stock price crashes not only damage investor welfare, but also interfere the sustainable development of capital market. This paper proposes and examines a new explanation for the management’s bad news hoarding behavior and the resulting risk of stock price crash. We concentrate on early-life traumatic experience. Specifically, we investigate whether CEOs who experienced the Great Famine in China during their early-life increase their firm-specific stock price crash risk. According to agency theory, CEOs conceal or delay bad news disclosure for a variety of personal benefits, such as position security, promotion, compensation growth, and empire building, etc. They also expect that such information can be “buried” over time (Jin and Myers, 2006; Kothari et al., 2009; Hermalin and Weisbach, 2012). When the cost of continuing to hide bad news will exceed the possible benefits of continuing to hide bad news, or objective conditions are no longer able to allow managers to continue to hide bad news, the hoarded bad news will be concentrated and instantly released into the capital market, causing a great impact on stock prices and finally forming a crash (Jin and Myers, 2006). On the basis of the bad news hoarding conjecture (Jin and Myers, 2006), there has been extensive research on the factors influencing stock price crash risk from different angles, such as information transparency (Hutton et al., 2009), excess management perks (Xu et al., 2014), religion (Li and Cai, 2016), accounting conservatism (Kim and Zhang, 2016), earnings smoothing (Chen et al., 2016), corporate social responsibility (Kim et al., 2014), institutional investors (An and Zhang, 2013), analyst (Xu et al., 2013), etc. Most of the prior studies focus on the influencing factors at the firm or environment levels, which ignores the impact of CEOs, who conceal bad news. While agency theory provides an important angle for explaining the bad news hoarding behavior of CEOs, it is based on an assumption that CEOs are homogeneous and self-interest, and thus ignores the heterogeneity and limited rationality of CEOs due to different personal characteristics or experiences. Due to the limitation of agency theory, scholars have used upper echelons theory and behavioral financial theory to study the impact of managers’ personal characteristics on the stock price crash risk. These personal characteristics include gender (Li and Zeng, 2019), political connection (Lee and Wang, 2016), power (Mamun et al., 2020) and overconfidence (Kim et al., 2016).

Prospect theory (Kahneman and Tversky, 1979) has been widely used to explain individuals’ judgment and decision-making behaviors in uncertain situations. According to this theory, most people prefer to take risk when facing losses. Therefore, people do not hate risks but losses. In this paper, we attempt to introduce prospect theory into the theoretical framework of the information hoard conjecture (Jin and Myers, 2006). For CEOs, bad news within the firm represent losses, and timely disclosure of bad news implies that expected losses will be translated into actual losses. Therefore, CEOs tend to irrationally hide or delay disclosuring the bad news inside the firm to avoid established losses, which may lead to a higher stock price crash risk. According to prospect theory, we explore a potentially complementary theory of the information hoard conjecture (Jin and Myers, 2006). Our view is that even if there is no conflict of interest between a CEO and shareholders, in the real decision making situation, the behavior of hiding bad news will still occur when the CEO is faced with the bad news in the firm due to the limited rationality. Prior literature shows that traumatic experiences, especially during early-life, have significant and lasting effects on the decision-making behavior of individuals (Main et al., 1985; Kenneth et al., 2002; Feng and Johansson, 2018). Trauma psychology emphasizes that long-term and repeated catastrophic experiences in early-life can easily cause individuals’ psychological trauma, which can lead to anxiety and depression in their adulthood and change their risk perceptions (Covello et al., 2001). The imprinting effect indicates that the complex social and historical situation affects the individual’s perceptual system, and the key social and historical events occurring in individual’s sensitive period would shape their perceptual system (Suddaby et al., 2015). As one of the most serious disasters in human history, the Great Famine in China (1959–1961) caused millions of people, especially the aged and children, to die of starvation, malnutrition, and food shortage related diseases. The tragic memory of the Great Famine has left people with eternal psychological trauma. Studies reveal that people who experienced the Great Famine during their early-life are more inclined to save (Cheng and Zhang, 2011). Managers who experienced this famine are also conservative in corporate decision makings, such as less debts and investments, more cash holding, and fewer takeovers (Zhang, 2017; Feng and Johansson, 2018; Hu et al., 2019).

In this paper, we take the Great Famine in China as an external traumatic event which cannot be selected or controlled by human. Based on a large sample of A-share listed firms in China from 2007 to 2017, we investigate whether firm-specific stock price crash risk can be explained by CEOs’ early-life famine experiences. According to cohort effect, groups with similar ages show similar personalities and behavioral characteristics due to similar experiences, cultural backgrounds, and social environments. These factors can affect their future decision-making behavior (Cheng and Zhang, 2011; Malmendier et al., 2011; Feng and Johansson, 2018). Therefore, we partition the CEOs into four cohorts according to their ages during the Great Famine: infancy or unborn, infantile, childhood or adolescence and adulthood. Since childhood and adolescence are the most critical stages in recognizing and understanding the world, preserving permanent memory, and forming character (Zhang, 2017), we focus on this cohort. According to prior studies (Cheng and Zhang, 2011; Zhang, 2017; Feng and Johansson, 2018), we define this stage as early-life. The results indicate that firms with CEOs who experienced the Great Famine during early-life have a higher stock price crash risk. The more severe and prolonged the Great Famine they experienced, the greater the effect of famine experience on crash risk. The step-by-step test method (Baron and Kenny, 1986) is used to further test the mediating effect of bad news hoarding behavior.

Our study contributes to the existing literature in the following ways. First, previous studies on stock price crash risk are based on the assumption that managers are homogenous or absolutely rational. This study breaks through this research hypothesis. The study analyses the path through that the compensation psychology and irrational defensive psychology caused by early-life Great Famine experience trigger CEOs bad news hoarding behavior. According to the Prospect Theory, the study explains that even if CEOs are not self-interested, they also tend to conceal bad news, leading to a higher crash risk, because of their bounded rationality and cognitive deviation caused by psychological trauma. CEOs in our paper are no longer limited to a single assumption of human nature, but real and comprehensive. In the complex judgment and decision-making process, they are characterized by both self-interest and bounded rationality. Our study inherits and develops the information hiding conjecture which is based on economic people assumption, and deepens the understanding of the reason of stock price crash risk. We provide a new theoretical explanation for the cause of stock price crash risk. Second, prior studies have focused on the impact of executives’ characteristic on stock price crash risk, such as gender (Li and Zeng, 2019), political connection (Lee and Wang, 2016), overconfidence (Kim et al., 2016), and power (Mamun et al., 2020). Traumatic experience is an important but often overlooked manager trait. This paper enriches the influencing factors of stock price crash risk from the view of managers’ heterogeneous characteristics. Third, the findings confirm that distant past experiences, which may form people’s cognitive and personality, have a substantial and lasting impact on managers’ behaviors. Compared to prior studies (Zhang, 2017; Feng and Johansson, 2018; Hu et al., 2019), what we have in common is respect for the fact that the Great Famine experience changed people’s perception of risk, and the famine CEOs were more risk-averse and lose-averse. However, the point of this paper is that just because the famine CEOs are afraid of risk doesn’t mean their firms have lower stock price crash risk. Inversely, afraid of taking risks and losses, famine CEOs tend to hide bad news, leading to a higher crash risk. We explain this bounded rationality behavior, break through the economic person hypothesis which is the premise of information hiding conjecture. Our findings add new evidence on the economic consequences of managers’ traumatic experiences in the field of stock price crash risk research.

## Institutional Background

In 1958, under the guidance of the “left” concept in China, the “Great Leap Forward” movement began to sweep across the country. Guided by the instruction “focus on industry instead of agriculture,” many rural labors were mobilized to the industrial sector, which had a serious impact on agricultural production. In addition, the strict implementation of the “unified purchase and selling” policy required farmers to comply the food procurement task before retaining their own food for living, seeds, and feed grains. Officials at all levels, who were influenced by radicalism, lied and reported high yields, which led to the escalation of food procurement tasks. The food in the hands of farmers was continuously levied, and even the food for living was difficult to preserve (Kung and Lin, 2003; Kung and Chen, 2011). This movement completely violated the economic laws. Thus, the large-scale movement of production that aimed to free China from poverty brought a disaster to this country. In 1958 the death rate reached 2.54%, whereas the faulty policies of government lasted for 3 years. Take Anhui Province as an example, the official death rate in 1960 reached to 6.86%, whereas that in 1957 was 0.91%.

The Great Famine is one of the largest and the serious recorded famine in human history. Although the exact data on the death caused by the famine has not been officially announced, previous studies reveal that the deaths estimated are 23 million (Peng, 1987), 27 million (Coale, 1981), or 29.5 million (Ashton et al., 1984). This tragic memory has left people with eternal psychological trauma, especially the ones witnessed the death of their families and friends. The Great Famine, therefore, provides a unique context that the CEOs with a traumatic experience during their early-life are investigated.

## Literature Review and Hypotheses Development

### Literature Review

Managers play a pivotal role in the decision-making process of a firm (Hambrick and Mason, 1984), specially, in the in the information disclosure decision (Soo and Hoon, 2017). With the deepening of research, scholars have gradually realized the important influence of managers’ personal characteristics on firm’s stock price crash risk. Powerful CEOs increase firm’s crash risk (Mamun et al., 2020). Firms with overconfident CEOs are associated with higher stock price crash risk (Kim et al., 2016). However, there are different results of the same influencing factors. Take gender, for example, some scholars have found that female CEOs could reduce the firm’s stock price crash risk significantly, while female CFO didn’t affect significantly (Li and Liu, 2012). Some scholars found a significant negative effect of female CFOs on crash risk, while this effect was not significant for CEOs (Li and Zeng, 2019). Take political connection, for example, some scholars argued that if a firm’s CEO or board chairperson has political experience, the firm has lower crash risk (Luo et al., 2016). However, some scholars revealed that the crash risk of firms with more political connection directors is higher (Lee and Wang, 2016). We infer that the cause of such contradictions may be the omission of some important variables that affect the psychology and behavior of managers.

The argument that experiences shape and influence human behavior has been supported by the multidisciplinary fields of psychology and sociology. Individuals are most affected by early-life exposure to disaster events (Elder, 1999). As for the impact of early traumatic experience on individuals, scholars mainly study from two aspects: the impact of early traumatic experience on family behavior and firm behavior. The Great Famines that happened long ago influence households’ consuming-saving decision (Cheng and Zhang, 2011). Those experienced more severe famine in their childhood are less likely to be self-employed because of their risk preference altered by famine experience, and they are also less willing to participate in financial market (Wang et al., 2015). When individuals who have experienced traumatic events during their early-life become managers of a firm, the impact of their traumatic experiences is not limited to the impact on family behavior, but the impact on firm behavior. CEOs who experienced the Great Depression during their early-life prefer internal financing to debt (Malmendier et al., 2011). Experiencing disasters of different severity has different effects on CEOs. CEOs who experienced natural disasters without extremely negative consequences appear to be desensitized to risk and lead firms that behave more aggressively. Conversely, CEOs who witnessed the extreme downside potential of natural disasters behave more conservatively when at the helm of a firm (Bernile et al., 2017). CEOs exposed to the war in their early-life tend to be conservative in corporate policies, especially those who have witnessed large-scale massacres (Choi et al., 2020). Scholars have focused traumatic events on the Great Famine in China from 1959 to 1961. The general consensus is that CEOs living through intense famines during early-life are conservative and risk aversion. Their firms have small debts, hold cash, and perform few takeovers.

### Hypotheses Development

Literature in psychology shows that the basis of human action comes from people’s memory of past knowledge and experience. Early feelings, impressions, memories, emotions, and knowledge play a critical role in the formation of people’s psychological orientation and personality (Holman and Silver, 1998). To a certain extent, people are the products of the social life they live in. Everyone belongs to a specific peer group; that is, the human group born in the same era and region. Many major social events, such as wars, economic depression, famines, epidemics and natural disaster have similar effects on the members of a particular peer group (Cheng and Zhang, 2011; Malmendier et al., 2011; Cameron and Shah, 2015; Bernile et al., 2017; Cassar et al., 2017; Feng and Johansson, 2018; Choi et al., 2020). Psychologists define this influence effect as cohort effect, which considers that similar age groups show similar personality and behavior characteristics due to similar experience and social environment. The painful experience and terrible memory of the Great Famine left a lasting scar on the generation who had experienced during early-life. We suppose that early-life Great Famine experience functions in two ways to enhance CEOs’ bad news hoarding behavior, which lead to a higher crash risk.

Firstly, the early-life Great Famine experience caused compensation psychology. Compensation, a kind of psychological defense mechanism, means that when individuals cannot achieve their goals, they can make up for these deficiencies in other ways to alleviate anxiety and build up self-esteem (Nagar, 1999). People often seek satisfaction and compensation in later life for extreme lack of material experience in the Great Famine. Some scholars confirmed that famine often leads to malnutrition in childhood and excessive eating and drinking in adulthood (Gluckman et al., 2005). We infer that this compensatory mentality makes CEOs who experienced the Great Famine during early-life more motivated to pursue promotion, pay growth, and empire building.

Second, severe traumatic memories of the famine caused irrational defensive psychology. Long-term and multiple disasters will strongly increase the psychological fear and the feeling of the uncertainty of expectations. Such individuals are more loss-averse, more cautious and conservative when making decisions (Zhang, 2017; Feng and Johansson, 2018). People witnessed various tragedies and even their own lives were in danger during the Great Famine, which could cause psychological trauma. Because of the fear of experiencing that tragedy again, people instinctively flee, forming an irrational defensive psychology. This irrational defensive psychology makes CEOs feel insecure and disgust with losses more intensely.

According to bad news hoarding conjecture, self-interested CEOs conceal or delay bad news disclosure for a variety of personal benefits. CEOs’ motivation to hide bad news will be heightened under the influence of traumatic psychology caused by living through the Great Famine. However, the rational economic behavior is only an ideal state, the behavior of economic individuals will be influenced by complex motives, and the bounded rational economic behavior is the normal state of the individuals (Simon, 1982). According to prospect theory (Kahneman and Tversky, 1979), people’s judgment and decision making under uncertain conditions are bounded rationality and biased. In the gain area, individuals are risk averse, they prefer ensured gains to potential losses. While, they pursuit risks, prefer uncertain gains, and hate established losses in the loss area. Actually, they do not hate risks but losses. For CEOs, bad news within firms represents expected losses, and timely disclosure implies that expected losses will become established losses. Therefore, CEOs hide or postpone bad news disclosure in the loss area. The CEOs’ traumatic experience of Great Famine during early-life exacerbated their cognitive bias to seek risk in the face of loss. Psychology suggests that motivation dominates behavior. The motivations abovementioned make CEOs who experienced the Great Famine during early-life inclined to hide bad news, leading to higher stock price crashes. Based on this, our hypothesis is following.

Hypothesis: Firms with CEOs who experienced the Great Famine during early-life have higher stock price crash risk.

## Research Design

### Sample Selection and Data Sources

The initial sample used in this paper consists of all Chinese A-share listed companies from 2007 to 2017. We screen the sample and exclude (1) financial firms; (2)firms with fewer than 30 trading weeks of stock return data in a fiscal year; (3) samples whose CEOs are of foreign, Hong Kong, or Taiwan nationality because the Great Famine only happened in Chinese mainland; and (4) observations with incomplete data. The study’s final sample consists of 13,887 firm-year observations. To mitigate the effects of outliers, we winsorize continuous variables at the 1% level in both tails. Our data are obtained from CSMAR database. Missing values are manually collected from their annual reports and the Great Famine data are from the National Bureau of Statistics of China.

### Variables and Model Specifications

#### Measurement of Stock Price Crash Risk

Following previous studies (Kim et al., 2016), we first estimate firm-specific weekly returns for each firm and year to examine firm-specific return crashes.


(1) $$\begin{array}{c} R_{i,t}=\alpha_{i}+\beta_{1i}R_{m,t-2}+\beta_{2i}R_{m,t-1}+\beta_{3i}R_{m,t}+\beta_{4i}R_{m,t+1}+\beta_{5i}R_{m,t+2}\\ +\beta_{6i}R_{d,t-2}+\beta_{7i}R_{d,t-1}+\beta_{8i}R_{d,t}+\beta_{9i}R_{d,t+1}+\beta_{10i}R_{d,t+2}+\varepsilon_{i,t} \end{array}$$

where *R*_*i,t*_ is the return that considers the yields on cash dividend reinvestment on stock *i* in week *t*, *R*_*m,t*_ is the value-weighted market return in week *t*, and *R*_*d,t*_ is the value-weighted industry (*d*, to which firm *i* belongs) return. The firm-specific weekly returns for firm *i* in week *t* are measured by *W*_*i*,*t*_ = *Ln*(1 + ε_*i*,*t*_), where ε_*i*,*t*_ is the residual in Eq. (1). On the basis of eliminating the influence of market, this method further eliminates the influence of the industry, making the firm-specific weekly return pure. Then we use two measures of crash risk proxy by *NCSKEW* and *DUVOL* to provide robust conclusion.


(2) $${\mathrm{NCSKEW}}_{i,t}=-\left[n{\left(n-1\right)}^{\frac{3}{2}}\sum W_{i,t}^{3}\right]/\left[\left(n-1\right)\left(n-2\right){\left(\sum W_{i,t}^{2}\right)}^{\frac{3}{2}}\right],$$

where *n* is the number of trading weeks of stock *i* in year *t*. A high value of *NCSKEW* means a high skewness coefficient, indicating a great crash risk.


(3) $${\mathrm{DUVOL}}_{i,t}=\log\left\{\left[\left(n_{\mathrm{up}}-1\right)\sum_{\mathrm{down}}W_{i,t}^{2}/\left(n_{\mathrm{down}}-1\right)\sum_{\mathrm{up}}W_{i,t}^{2}\right]\right\},$$

where *n*_*up*_ and *n*_*down*_ are the number of up and down weeks in year *t*, respectively. A high value of *DUVOL* indicates a left-skewed distribution, which means that the stock has a great crash risk.

#### Measures of Early-Life Great Famine Experiences

**(1) Cohort**

Cohort refers to the set of people who experienced the same events in the same period. The academic community has generally adopted the cohort effect to examine the impact of a specific experience on people in a certain period (Malmendier and Nagel, 2009; Cheng and Zhang, 2011; Malmendier et al., 2011; Feng and Johansson, 2018). It is an effective way to analyze the impact of social changes on individual life course. The famine covered almost all provinces and regions of mainland China, and the household registration system at that time largely limited the flow of people. Therefore, the birth cohort during the Great Famine can measure and characterize CEOs’ experience.

In accordance with Erikson’s theory of personality development (Domino and Affonso, 1990), this study classifies CEOs in the infant (less than 3 years old) or unborn stage during the Great Famine as Cohort 1; infantile period (3 to 6 years old) as Cohort 2; childhood and adolescence stage (7 to 17 years old) as Cohort 3; and adulthood stage (larger than 18 years old) as Cohort 4. The classifications are presented in Table 1. According to developmental psychology, among the four cohorts, childhood and adolescence are the most critical period in knowing and understanding the world, forming personality, and preserving permanent memory (Tulving, 2002). The early-life that we focus on in this paper also refers to this period (Cohort 3).

**TABLE 1:** Classification of the birth cohort.

Birth cohort	Life cycle	Birth year	Age during 1959–1961	Obs.
Cohort 1	Infancy or unborn	(1958,	, 3)	11118
Cohort 2	Infantile	(1954, 1958]	[3, 7)	1656
Cohort 3	Childhood or adolescence	(1941, 1954]	[7, 18)	1095
Cohort 4	Adulthood	, 1941]	[18,	18

**(2) Famine Severity**

The intensity of the experience has a greater impact on CEOs’ risk tolerance than the event itself (Bernile et al., 2017). A large difference exists in the famine severity in different provinces during the Great Famine. In the relatively serious case of Anhui Province, the official mortality data in 1960 was 6.86%, whereas the death rate in Shanghai, which was hardly hit, was only 0.68%, similar to normal years. This structural difference is important for understanding famine.

**FIGURE 1:**
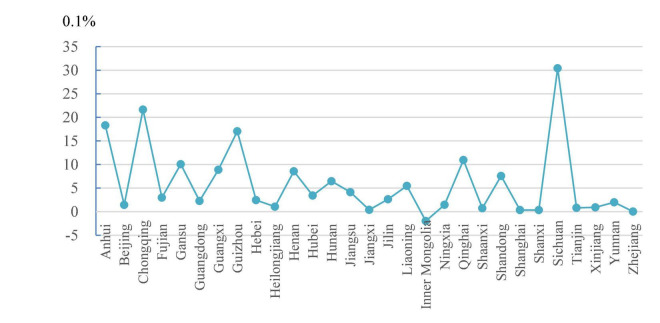
Famine severity across provinces.

In accordance with existing research practices (Chen and Zhou, 2007; Cheng and Zhang, 2011), we define *Famine_severity* measured by excess death rate (EDR) as an indicator of the famine severity in the province. The larger the index, the higher the famine severity. The EDR in each province is defined as the average death rate of the 3 year famine in the province (1959–1961) minus the average death rate of the 5 year pre-famine period (1954–1958). Figure 1 and Table 2 present the EDRs during the Great Famine in different provinces, highlighting the differences among provinces. Sichuan, Chongqing, Anhui, and Guizhou are the most affected by the Great Famine, whereas Inner Mongolia, Zhejiang, Shanxi, Jiangxi, and Shanghai are the least affected.

**TABLE 2:** Death rates and famine severity across provinces (unit: 0.1%).

Province	1954	1955	1956	1957	1958	1959	1960	1961	EDR
Sichuan	8.4	9.2	10.4	12.1	25.2	47	54	29.4	30.4
Chongqing	13.3	11.7	11.3	11	16.1	31.5	44.9	26.5	21.6
Anhui	16.6	11.8	14.3	9.1	12.3	16.7	68.6	8.1	18.3
Guizhou	8.8	8.1	7.5	8.8	13.7	16.2	45.4	17.7	17.1
Qinghai	13.3	14.1	9.4	10.4	13	16.6	40.7	11.7	11
Gansu	11.6	11.9	10.8	11.3	21.1	17.4	41.3	11.5	10.1
Guangxi	15.2	14.6	12.5	12.4	11.7	17.5	29.5	19.5	8.9
Henan	13.3	11.8	14	11.8	12.7	14.1	39.6	10.2	8.6
Shandong	11.7	13.7	12.1	12.1	12.8	18.2	23.6	18.4	7.6
Hunan	17.5	16.4	11.5	10.4	11.7	13	29.4	17.5	6.5
Liaoning	8.6	9.4	6.6	9.4	6.6	11.8	11.5	17.5	5.5
Jiangsu	12.2	11.8	13	10.3	9.4	14.6	18.4	13.4	4.1
Hubei	15.9	11.6	10.8	9.6	9.6	14.5	21.2	9.1	3.4
Fujian	10.9	8.9	8.4	7.9	7.5	7.9	15.3	11.9	3
Jilin	10.4	9.9	7.5	9.1	9.1	13.4	10.1	12	2.6
Hebei	12.1	11.6	11.3	11.3	10.9	12.3	15.8	13.6	2.5
Guangdong	11.2	10.6	11.1	8.4	9.2	11.1	15.2	10.8	2.3
Yunnan	16.7	13.7	15.2	16.3	21.6	18	26.3	11.8	2
Ningxia	13.1	10.2	10.6	11.1	15	15.8	13.9	10.7	1.5
Beijing	8.6	9.5	7.7	8.2	8.1	9.7	9.1	10.8	1.4
Heilongjiang	11.1	11.3	10.1	10.5	9.2	12.8	10.6	11.1	1.1
Xinjiang	16.8	14.4	14.2	14	13	18.8	15.7	11.7	0.9
Tianjin	9.3	9.9	8.8	9.4	8.7	9.9	10.3	9.9	0.8
Shaanxi	11	10.5	9.9	10.3	11	12.7	12.3	8.8	0.7
Jiangxi	14.2	16.2	12.5	11.5	11.3	13	16.1	11.5	0.4
Shanghai	7.1	8.1	6.8	6	5.9	6.9	6.8	7.7	0.4
Shanxi	14.7	12.9	11.6	12.7	11.7	12.8	14.2	12.2	0.3
Zhejiang	13.4	12.6	9.5	9.3	9.2	10.8	11.9	9.8	0.03
Inner Mongolia	20.9	11.4	7.9	10.5	7.9	11	9.4	8.8	-2

*Source: National Bureau of Statistics.*

**(3) Famine Duration**

Although the official time for the Great Famine is 1959 to 1961, the actual durations for each province are different. The national Great Famine outbreak began in spring 1959. Sichuan, Gansu, Anhui, and Yunnan indicated a dramatic increase in mortality in winter 1958. In 1962, the Great Famine stopped in most provinces of the country, but that in Sichuan and Chongqing did not end until 1963. Considering the differences in the famine duration in different regions, we define variable *Famine_duration* as another proxy variable of famine severity. The provinces with famine lasting for more than 3 years are assigned a value of 1, whereas those with famine lasting 3 years or less are assigned a value of 0. Table 3 presents the durations of famine in each province.

**TABLE 3:** Great Famine period across provinces.

Province	Great Famine period	Province	Great Famine period
Anhui	1958–1961	Liaoning	1959–1962
Beijing	1958–1961	Inner Mongolia	1959, 1961
Fujian	1959–1961	Ningxia	1959–1961
Gansu	1959–1961	Qinghai	1959–1961
Guangdong	1959–1961	Shandong	1958–1961
Guangxi	1959–1961	Shanxi	1959–1961
Guizhou	1959–1961	Shaanxi	1960–1961
Hebei	1959–1961	Shanghai	1958–1961
Henan	1958–1961	Sichuan	1959–1962
Heilongjiang	1961	Tianjin	1959–1962
Hubei	1959–1961	Xinjiang	1959, 1961
Hunan	1959–1961	Yunnan	1959–1961
Jilin	1959, 1961	Zhejiang	1959–1961
Jiangsu	1958–1961	Chongqing	1959–1962
Jiangxi	1959–1961		

*Source: Reprinted in Peng (1987).*

#### Model Specifications

The basic regression model is as follows:


(4) $${\mathrm{CrashRisk}}_{i,t+1}=\alpha_{0}+\sum_{c=2}^{4}\beta_{c}{\mathrm{Cohort}}_{i,c}+\gamma \times {\mathrm{Controls}}_{i,t}+\varepsilon_{i,t,}$$

where *CrashRisk* refers to the firm-specific stock price crash risk proxied by *NCSKEW* or *DUVOL*. The dependent variable is measured in year t+1, whereas the independent variables are measured in year t. The main independent variables are *Cohort_*i*_,_*c*_*.

In accordance with prior studies (Kim et al., 2016; Luo et al., 2016), we include a set of control variables, including the lagged *NCSKEW/DUVOL*, firm value (*SIZE*), the weekly return volatility (*SIGMA*), the detrended turnover of each stock (*DTURN*), the past returns (*RET*), the market-to-book ratio (MB), the returns on equity (*ROE*), firm leverage (LEV), the detrended average monthly stock turnover (*OTUM*), firm ownership (*SOE*), the information transparency variable (*ABACC*) and the accounting conservatism score (*C_SCORE*). Personal characteristics are one of the important factors that affect personal experience and corporate outcomes (Benmelech and Frydman, 2015). Upper echelons theory (UET), which was developed by Hambrick and Mason (1984), posits that a manager’s characteristics shape organizational outcomes. UET posits that top managers’ characteristics, such as age, education or tenure, affect their decisions regarding strategy and structure and directly affect a firm’s strategic choices and performance. Furthermore, UET emphasizes that managers will make decisions based on their cognitive characteristics. The human capital relates strongly to performance and should be invested and retained. To control for the personal characteristics of CEOs, we consider two variables: *GENDER*, the gender of the CEO, where equals to 1 for male and 0 otherwise, and *EDU*, proxied by CEO’s educational attainment, where equals to 1 if the CEO holds master or doctoral degree and 0 otherwise. To separate the effect of famine severity from province characteristics, we control for the place of registration by using the marketization index of China’s province. Moreover, we control for the influence of year and industry. The definitions of detailed variables can be found in Appendix A.

Before the 1959-1961 Great Famine, no one predicted the occurrence of famine. Famine is an exogenous shock to people and is therefore a random natural experiment. The Great Famine swept across the country, but the severity of the famine varied from province to province, providing a good opportunity to study the relationship between traumatic experiences during early-life and decision-making behavior later. Following prior study (Chen and Zhou, 2007), we establish a difference-in-difference (DID) model using different famine severities and different birth cohorts.


(5) $$\begin{array}{c} {\mathrm{CrashRisk}}_{i,t+1}=\alpha_{0}+\sum_{c=2}^{4}\beta_{c}{\mathrm{Cohort}}_{i,c}+\varphi_{s}\times \mathrm{Famine}\_{\mathrm{severity}}_{j}\\ +\sum_{c=2}^{4}\delta_{c}{\mathrm{Cohort}}_{i,c}\times \mathrm{Famine}\_{\mathrm{severity}}_{j}+\gamma \times {\mathrm{Controls}}_{i,t}+\varepsilon_{i,t}. \end{array}$$

where θ, the interaction coefficient between famine duration and birth cohort, represents the impact of CEOs in different birth cohorts experiencing different famine durations on firms’ crash risk.

## Empirical Results

### Descriptive Statistics

According to Table 4, the mean values of *NCSKEW_t+_*_1_ and *DUVOL_*t*+_*_1_ are −0.251 and −0.171, respectively. According to Table 5, consistent with our prediction, firms with CEOs who experienced the Great Famine during their early-life (*Cohort* 3_*t*_) have higher crash risk.

**TABLE 4:** Descriptive statistics.

Variable	N	Mean	Median	p25	p75	S.D.
*NCSKEW*_*t*+1_	13887	–0.251	–0.242	–0.847	0.362	0.945
*DUVOL*_*t*+1_	13887	–0.171	–0.172	–0.723	0.379	0.835
*Cohort 1*_*t*_	13887	0.801	1	1	1	0.400
*Cohort 2*_*t*_	13887	0.119	0	0	0	0.324
*Cohort 3*_*t*_	13887	0.079	0	0	0	0.270
*Cohort 4*_*t*_	13887	0.001	0	0	0	0.036
*NCSKEW*_*t*_	13887	–0.280	–0.261	–0.844	0.318	0.919
*DUVOL*_*t*_	13887	–0.196	–0.192	–0.725	0.338	0.809
*SIZE*_*t*_	13887	22.100	21.98	21.22	22.88	1.303
*LEV*_*t*_	13887	0.506	0.510	0.351	0.659	0.209
*ROE*_*t*_	13887	0.065	0.069	0.024	0.126	0.150
*MB*_*t*_	13887	2.028	1.444	0.795	2.487	1.976
*OTUM*_*t*_	13887	–0.0690	–0.001	–0.290	0.180	0.375
*RET*_*t*_	13887	0.023	0.016	–0.013	0.054	0.054
*SIGMA*_*t*_	13887	0.145	0.132	0.102	0.174	0.060
*ABACC*_*t*_	13887	0.090	0.069	0.044	0.107	0.080
*C_SCORE*_*t*_	13887	–0.428	–0.097	–0.670	0	0.554
*SOE*_*t*_	13887	0.574	1	0	1	0.495
*Registry*_*t*_	13887	7.354	7.410	6.100	8.850	1.850
*GENDER*_*t*_	13887	0.948	1	1	1	0.222
*EDU*_*t*_	13887	0.417	0	0	1	0.493

**TABLE 5:** Univariate analysis results.

Cohort	Obs.	*NCSKEW*_*t*+1_		*DUVOL*_*t*+1_	
		Mean	Median	Mean	Median
*Cohort 3*_*t*_	1095	–0.181	–0.152	–0.125	–0.116
Others	12792	–0.257	–0.253	–0.175	–0.177
Difference		0.076**	0.101**	0.050*	0.061**

* and ** indicate statistical significance at the 10% and 5% significance level.

### Effects of Early-Life Great Famine Experiences on Stock Price Crash Risk

Table 6 presents the findings of the regression analysis. Consistent with the univariate analysis results, the coefficients associated with *Cohort* 3 are positive and significant at the 1% and 5% levels. This finding supports our hypothesis, that is, firms with CEOs who experienced the Great Famine during early-life have higher stock price crash risk. The influences of control variables on crash risk are generally consistent with prior research.

**TABLE 6:** Regression results of Great Famine experiences on firms’ stock price crash risk.

	(1) *NCSKEW*_*t*+1_	(2) *DUVOL*_*t*+1_
*Cohort 2*_*t*_	−0.003	−0.010
	(–0.13)	(–0.46)
*Cohort 3*_*t*_	0.082***	0.067**
	(2.74)	(2.53)
*Cohort 4*_*t*_	−0.065	0.111
	(–0.30)	(0.58)
*NCSKEW*_*t*_	0.026***	
	(2.66)	
*DUVOL*_*t*_		0.021**
		(2.04)
*SIZE*_*t*_	0.038***	0.044***
	(4.20)	(5.50)
*LEV*_*t*_	0.009	−0.032
	(0.20)	(–0.81)
*ROE*_*t*_	−0.008	−0.050
	(-0.15)	(–0.98)
*MB*_*t*_	0.043***	0.034***
	(7.77)	(6.88)
*OTUM*_*t*_	−0.083***	−0.098***
	(–2.88)	(–3.83)
*RET*_*t*_	3.962***	3.813***
	(10.50)	(10.88)
*SIGMA*_*t*_	−0.488**	−0.089
	(–2.29)	(–0.47)
*ABACC*_*t*_	0.304***	0.272***
	(2.79)	(2.82)
*C_SCORE*_*t*_	−3.857***	−4.541***
	(–10.98)	(–14.61)
*SOE*_*t*_	0.028	0.036**
	(1.55)	(2.27)
*Registry*_*t*_	−0.019***	−0.017***
	(–3.90)	(–4.06)
*GENDER*_*t*_	0.010	−0.003
	(0.28)	(–0.09)
*EDU*_*t*_	−0.012	−0.017
	(–0.76)	(–1.17)
*Constant*	−3.955***	−4.521***
	(−12.03)	(–15.55)
Year	YES	YES
Industry	YES	YES
N	13887	13887
Adj R^2^	0.061	0.058

T-values are reported in parentheses. ** and *** indicate statistical significance at the 5% and 1% significance level, respectively.

### Famine Severity

As mentioned in Table 2, the severity of the Great Famine varied quite significantly across provinces, which provides us with a rare opportunity to study the effect of the Great Famine experience during early-life on bad news hoarding behavior in later years. Table 7 displays the regression results using Eq. (5). The interaction variable for *Famine_severity* and *Cohort* 3 are significantly positive at the 5% and 10% levels. This finding supports our hypothesis. Firms with CEOs who experienced the Great Famine during early-life have higher stock price crash risk. The higher the severity of their famine experience, the higher the stock price crash risk.

**TABLE 7:** Difference-in-difference estimation by using different cohorts and famine severities.

	(1) *NCSKEW*_*t*+1_	(2) *DUVOL*_*t*+1_
*Cohort 2*_*t*_	0.027	0.015
	(0.49)	(0.31)
*Cohort 3*_*t*_	0.112*	0.105**
	(1.94)	(2.08)
*Cohort 4*_*t*_	0.268	0.306
	(0.37)	(0.49)
*Famine_severity*_*t*_	−0.004	−0.002
	(–1.36)	(–0.85)
*Cohort 2*_*t*_ *Famine_ severity_*t*_	−0.002	−0.004
	(–0.28)	(–0.66)
*Cohort 3*_*t*_ *Famine_ severity_*t*_	0.017**	0.011*
	(2.14)	(1.69)
*Cohort 4*_*t*_ *Famine_ severity_*t*_	−0.063	−0.034
	(–0.41)	(–0.25)
*NCSKEW*_*t*_	0.021	
	(1.18)	
*DUVOL*_*t*_		0.012
		(0.65)
*SIZE*_*t*_	0.039**	0.047***
	(2.49)	(3.46)
*LEV*_*t*_	−0.020	−0.068
	(–0.23)	(–0.87)
*ROE*_*t*_	−0.074	−0.143
	(–0.67)	(–1.47)
*MB*_*t*_	0.051***	0.036***
	(4.90)	(3.99)
*OTUM*_*t*_	−0.079	−0.118**
	(–1.50)	(–2.56)
*RET*_*t*_	3.518***	3.611***
	(5.31)	(5.90)
*SIGMA*_*t*_	−0.589	−0.368
	(–1.52)	(–1.08)
*ABACC*_*t*_	0.260	0.142
	(1.25)	(0.78)
*C_SCORE*_*t*_	−3.576***	−4.346***
	(–5.60)	(–7.77)
*SOE*_*t*_	0.030	0.023
	(0.86)	(0.77)
*Registry*_*t*_	−0.024**	−0.025***
	(–2.42)	(–2.83)
*GENDER*_*t*_	0.058	0.041
	(0.84)	(0.68)
*EDU*_*t*_	−0.020	−0.015
	(–0.64)	(–0.54)
*Constant*	−3.555***	−4.218***
	(–6.07)	(–8.22)
Year	YES	YES
Industry	YES	YES
N	4118	4118
Adj R^2^	0.070	0.064

T-values are reported in parentheses. *, **, and *** indicate statistical significance at the 10%, 5%, and 1% significance level, respectively.

### Famine Duration

As mentioned in Table 3, the Great Famine lasts for different periods across provinces, which also provides us with the research opportunities to construct a causal relationship between the famine experience during early-life and bad news hoarding behavior in later years. Eq. (6) constructed DID model with different famine duration and different birth cohort.

Table 8 displays the regression results using Eq. (6). The interaction variable for *Famine_ duration* and *Cohort* 3 are significantly positive at the 5% and 10% levels. This finding supports our hypothesis. Firms with CEOs who experienced the Great Famine during early-life have higher stock price crash risk. The longer the duration of their famine experience, the higher the stock price crash risk.

**TABLE 8:** Difference-in-difference estimation by using different cohorts and famine durations.

	(1) *NCSKEW*_*t*+1_	(2) *DUVOL*_*t*+1_
*Cohort 2*_*t*_	0.064	0.019
	(1.18)	(0.40)
*Cohort 3*_*t*_	0.073	0.083
	(1.11)	(1.44)
*Cohort 4*_*t*_	−0.281	−0.226
	(–0.31)	(–0.28)
*Famine_duration*_*t*_	0.020	0.021
	(0.56)	(0.68)
*Cohort 2*_*t*_ *Famine_ duration_*t*_	−0.112	−0.051
	(–1.25)	(–0.65)
*Cohort 3*_*t*_ *Famine_ duration_*t*_	0.220**	0.150*
	(2.36)	(1.84)
*Cohort 4*_*t*_ *Famine_ duration_*t*_	0.302	0.417
	(0.32)	(0.50)
*NCSKEW*_*t*_	0.023	
	(1.25)	
*DUVOL*_*t*_		0.013
		(0.69)
*SIZE*_*t*_	0.037**	0.046***
	(2.35)	(3.37)
*LEV*_*t*_	0.004	−0.047
	(0.05)	(–0.60)
*ROE*_*t*_	−0.066	−0.136
	(–0.59)	(–1.40)
*MB*_*t*_	0.050***	0.036***
	(4.87)	(3.94)
*OTUM*_*t*_	−0.072	−0.116**
	(–1.37)	(–2.52)
*RET*_*t*_	3.513***	3.614***
	(5.30)	(5.91)
*SIGMA*_*t*_	−0.550	−0.344
	(–1.42)	(–1.02)
*ABACC*_*t*_	0.251	0.136
	(1.21)	(0.75)
*C_SCORE*_*t*_	−3.529***	−4.317***
	(–5.53)	(–7.73)
*SOE*_*t*_	0.021	0.016
	(0.60)	(0.54)
*Registry*_*t*_	−0.023**	−0.024***
	(–2.31)	(–2.82)
*GENDER*_*t*_	0.068	0.047
	(0.99)	(0.79)
*EDU*_*t*_	−0.018	−0.014
	(–0.58)	(–0.51)
*Constant*	−3.567***	−4.230***
	(–6.10)	(–8.26)
Year	YES	YES
Industry	YES	YES
N	4118	4118
Adj R^2^	0.071	0.065

T-values are reported in parentheses. *, **, and *** indicate statistical significance at the 10%, 5%, and 1% significance level, respectively.

### Mediating Effect of Bad News Hoarding Behavior

In Section “Literature Review and Hypotheses Development,” we analyzed that CEOs who experienced the Great Famine during early-life are more inclined to hide bad news under the action of compensation psychology and irrational defensive psychology, which leads to higher risk of stock price crash. We speculate that the behavior of hoarding bad news plays an important mediating effect between early famine experience and stock price crash risk. The step-by-step test method (Baron and Kenny, 1986) is used to analyze this mediating effect. Path models a, b, and c are set as follows:


(7a) $${\mathrm{CrashRisk}}_{i,t+1}=\alpha_{0}+\alpha_{1}\times {\mathrm{Cohort3}}_{i,t}+\alpha_{2}\times {\mathrm{Controls}}_{i,t}+\varepsilon_{i,t}$$

(7b) $$\mathrm{Bad}\mathrm{news}{\mathrm{hiding}}_{i,t}=\beta_{0}+\beta_{1}\times {\mathrm{Cohort3}}_{i,t}+\beta_{2}\times {\mathrm{Controls}}_{i,t}+\varepsilon_{i,t}$$

(7c) $$\begin{array}{c} {\mathrm{CrashRisk}}_{i,t+1}=\gamma_{0}+\gamma_{1}\times {\mathrm{Cohort3}}_{i,t}+\gamma_{2}\times \mathrm{Bad}\mathrm{news}{\mathrm{hiding}}_{i,t}\\ +\gamma \times {\mathrm{Controls}}_{i,t}+\varepsilon_{i,t} \end{array}$$

where α_1_ in Eq. (a) is the total effect of famine CEOs on stock price crash risk, β_1_ in Eq. (b) is the effect of famine CEOs on bad news hoarding, and γ_2_ in Eq. (c) is the effect of the mediating variable of bad news hoarding on stock price crash risk.

In China, China Securities Regulatory Commission and other regulatory authorities disclose listed companies that violate rules. These regulators give penalties, such as criticism, warning, condemnation, or fines, every year. A total of 1,724 observations were reported for violation among 13,887 observations. According to the announcement, listed companies’ violations include fictitious profits, false assets, false records, disclosure postponement, major omissions, false disclosures, etc. We consider that the violations mentioned above can proxy the firm’s hoarding bad news behavior. Such violations can be used as proxy variables to hide bad news. A dummy variable, *Violations*, is equal to 1 when the listed companies are punished for violation and 0 otherwise.

Table 9 presents the mediating effects of regression testing for whether famine CEOs increase firm-specific crash risk by hoarding bad news. In columns (1) and (4), the coefficient of *Cohort* 3 is 0.083 and 0.069 and significant at the 1% level, which suggests that famine CEOs increase firm-specific crash risk. In columns (2) and (5), the coefficient of *Cohort* 3 is positive, indicating that famine CEOs are likely to hoard bad news. When we add the mediating factor violations into columns (3) and (6), the coefficient of *Cohort* 3 decreases from 0.083 (0.069) to 0.075 (0.063) and its significance level decreases from the 1% to 5% level. We also used the Sobel test method to confirm the result, and the Sobel *z*-value is 2.166 (2.109), which is significant at the 5% level. There is a partial mediating effect of hoarding bad news between early-life famine experience of CEOs and stock price crash risk. We conclude that the mechanism that CEOs’ early-life traumatic famine experience affects the stock price crash risk is the behavior of hoarding bad news.

**TABLE 9:** Channel test: Bad news hoarding.

	(1)	(2)	(3)	(4)	(5)	(6)
	Path a	Path b	Path c	Path a	Path b	Path c
	*NCSKEW*_*t*+1_	Violations	*NCSKEW*_*t*+1_	*DUVOL*_*t*+1_	violations	*DUVOL*_*t*+1_
*Cohort 3*_*t*_	0.083***	0.282***	0.075**	0.069***	0.282***	0.063**
	(2.79)	(5.33)	(2.53)	(2.61)	(5.33)	(2.41)
*Violations*_*t*_			0.135***			0.089***
			(5.60)			(4.15)
*Controls*_*t*_	YES	YES	YES	YES	YES	YES
Year	YES	YES	YES	YES	YES	YES
Industry	YES	YES	YES	YES	YES	YES
N	13887	13792	13887	13887	13792	13887
Adj R^2^	0.061	0.069	0.063	0.059	0.069	0.060
Sobel Z			2.166**			2.109**

T-values are reported in parentheses. ** and *** indicate statistical significance at the 5% and 1% significance level, respectively.

### Robustness Checks

#### Controlling for Potential Bias Due to Local Culture

CEOs born in the same area may have similar risk preferences because of the same local culture (Chang et al., 2013). To further isolate the famine effect from the similar local culture effect, we design a new test using the CEOs who didn’t experience the Great Famine but were affected by similar local cultures as the control group. During 1959–1961, Hong Kong and Taiwan were neither affected by political influence nor suffered from the Great Famine. However, the people of Hong Kong and Taiwan share the same culture as China, especially with neighboring Guangdong and Fujian. Therefore, we use CEOs born in Guangdong and Fujian as the treatment group and those born in Hong Kong and Taiwan as the control group. *Exp* is a dummy variable that equals to 1 if the CEOs are born in Guangdong or Fujian and equals to 0 if they are born in Hong Kong or Taiwan. We focus on the interaction coefficient between *Cohort* 3 and *Exp*. This coefficient measures the estimated effects of CEOs having experienced Great Famine during early-life on firms’ crash risk.

Table 10 presents the results. The interaction coefficient between *Cohort* 3 and *Exp* is 1.196 (1.339), which is statistically significant at the 10% (5%) level. This finding suggests that firms with CEOs who experienced Great Famine during their early-life have high stock price crash risk.

**TABLE 10:** Robustness check: Controlling for potential bias due to local culture.

	(1) *NCSKEW*_*t*+1_	(2) *DUVOL*_*t*+1_
*Cohort 2*_*t*_	0.186	0.113
	(0.44)	(0.31)
*Cohort 3*_*t*_	−1.108	−1.225**
	(–1.63)	(–2.12)
*Cohort 4*_*t*_	0.000	0.000
	(.)	(.)
*Exp*_t_	0.149	−0.012
	(0.45)	(–0.04)
Cohort 2* Exp_t_	0.066	0.044
	(0.15)	(0.12)
Cohort 3* Exp_t_	1.196*	1.339**
	(1.71)	(2.24)
Cohort 4* Exp_t_	0.000	0.000
	(.)	(.)
*NCSKEW*_*t*_	−0.022	
	(–0.45)	
*DUVOL*_*t*_		−0.026
		(–0.51)
*SIZE*_*t*_	0.049	0.058
	(1.06)	(1.45)
*LEV*_*t*_	0.094	0.140
	(0.32)	(0.57)
*ROE*_*t*_	0.005	−0.085
	(0.01)	(–0.25)
*MB*_*t*_	0.052	0.040
	(1.58)	(1.45)
*OTUM*_*t*_	−0.371**	−0.365***
	(–2.54)	(–2.93)
*RET*_*t*_	3.799**	3.733**
	(1.97)	(2.14)
*SIGMA*_*t*_	0.103	−0.172
	(0.09)	(–0.17)
*ABACC*_*t*_	0.629	0.597
	(1.35)	(1.50)
*C_SCORE*_*t*_	−4.134*	−7.166***
	(–1.79)	(–3.64)
*GENDER*_*t*_	0.255	0.103
	(1.22)	(0.58)
*EDU*_*t*_	0.023	−0.000
	(0.25)	(–0.00)
*Constant*	−5.183**	−7.086***
	(–2.36)	(–3.78)
Year	YES	YES
Industry	YES	YES
N	617	617
Adj R^2^	0.074	0.052

T-values are reported in parentheses. *, **, and *** indicate statistical significance at the 10%, 5%, and 1% significance level, respectively.

#### Controlling for Potential Bias in the Difference-in-Difference Estimator

To control for potential bias in the DID estimator, we follow Chen and Zhou (2007) and reestimate the model using a sub-sample of CEOs who were born after the Great Famine. None of these CEOs were directly exposed to the famine. Thus, we expect that the Great Famine cannot produce consistent effects on crash risk for this sub-sample. We create four new cohorts for CEOs in accordance with the age classification of Cohorts 1 to 4. CEOs were born after 1982 are in *Cohort* 5; born during 1978 and 1982 are in *Cohort* 6; born during 1965 and 1978 are in *Cohort* 7; and born during 1962 and 1965 are in *Cohort* 8. Then, we run new regressions with these cohorts. To verify the reliability of model 5 and the results in Table 7, we focus on the interaction coefficient between *Cohort* 7 and *Famine_severity*. To verify the reliability of model 6 presented in Table 8, we pay attention to the interaction coefficient between *Cohort* 7 and *Famine_duration.*

According to Table 11, the results in columns (1) and (2) reveal that the interaction coefficient between *Cohort* 7 and *Famine_severity* is no longer significant. The findings in columns (3) and (4) indicate that the interaction coefficient between *Cohort* 7 and *Famine_duration* is also no longer significant. These results strongly support the argument that the DID estimator in Tables 7, 8 captures the effect of CEOs early-life experience in the Great Famine, rather than other omitted variables.

**TABLE 11:** Robustness check: Testing the assumption behind the DID estimation.

	(1)	(2)	(3)	(4)
	*NCSKEW*_*t*+1_	*DUVOL*_*t*+1_	*NCSKEW*_*t*+1_	*DUVOL*_*t*+1_
*Cohort 6*_*t*_	−0.720	−0.463	0.441	0.444
	(–0.61)	(–0.45)	(0.45)	(0.52)
*Cohort 7*_*t*_	−0.718	−0.536	0.512	0.486
	(–0.62)	(–0.53)	(0.53)	(0.58)
*Cohort 8*_*t*_	−0.756	−0.542	0.453	0.461
	(–0.65)	(–0.53)	(0.47)	(0.55)
*Famine _severity*_*t*_	−0.553	−0.457		
	(–0.78)	(–0.74)		
*Cohort 6*_*t*_ *Famine _severity_*t*_	0.530	0.398		
	(0.74)	(0.63)		
*Cohort 7*_*t*_ *Famine _severity_*t*_	0.549	0.455		
	(0.77)	(0.73)		
*Cohort 8*_*t*_ *Famine _severity_*t*_	0.553	0.456		
	(0.78)	(0.73)		
*Famine_duration*			1.053	0.868
			(0.77)	(0.73)
*Cohort 6*_*t*_ *Famine _duration_*t*_			−0.133	−0.150
			(–0.08)	(–0.10)
*Cohort 7*_*t*_ *Famine _duration_*t*_			−1.068	−0.875
			(–0.78)	(–0.73)
*Cohort 8*_*t*_ *Famine _duration_*t*_			−0.986	−0.834
			(–0.72)	(–0.70)
*NCSKEW*_*t*_	0.047*		0.046*	
	(1.81)		(1.79)	
*DUVOL*_*t*_		0.047*		0.046*
		(1.73)		(1.69)
*SIZE*_*t*_	0.026	0.035*	0.028	0.036*
	(1.10)	(1.66)	(1.17)	(1.71)
*LEV*_*t*_	0.023	−0.041	0.017	−0.044
	(0.17)	(–0.35)	(0.13)	(–0.37)
*ROE*_*t*_	−0.077	−0.091	−0.090	−0.102
	(–0.50)	(–0.67)	(–0.58)	(–0.76)
*MB*_*t*_	0.050***	0.038***	0.050***	0.038***
	(3.50)	(3.04)	(3.52)	(3.05)
*OTUM*_*t*_	−0.012	−0.081	−0.015	−0.082
	(–0.16)	(–1.20)	(–0.19)	(–1.21)
*RET*_*t*_	3.821***	4.059***	3.788***	4.027***
	(3.97)	(4.58)	(3.94)	(4.54)
*SIGMA*_*t*_	−1.244**	−0.971**	−1.210**	−0.938*
	(–2.22)	(–1.98)	(–2.17)	(–1.92)
*ABACC*	0.090	0.015	0.071	0.006
	(0.31)	(0.06)	(0.25)	(0.03)
*C_SCORE_t_*	−3.772***	−4.510***	−3.722***	−4.477***
	(–4.24)	(–5.79)	(–4.18)	(–5.75)
*SOE*_*t*_	0.023	0.051	0.014	0.045
	(0.44)	(1.13)	(0.26)	(0.97)
*Registry*_*t*_	−0.027*	−0.031**	−0.026*	−0.029**
	(–1.79)	(–2.29)	(–1.75)	(–2.22)
*GENDER*_*t*_	0.134	0.097	0.138	0.095
	(1.07)	(0.88)	(1.09)	(0.86)
*EDU*_*t*_	0.061	0.039	0.058	0.039
	(1.36)	(1.00)	(1.30)	(0.98)
*Constant*	−2.854**	−3.599***	−4.097***	−4.635***
	(–1.96)	(–2.83)	(–3.20)	(–4.13)
Year	YES	YES	YES	YES
Industry	YES	YES	YES	YES
N	2119	2119	2119	2119
Adj R^2^	0.065	0.057	0.066	0.058

T-values are reported in parentheses. *, **, and *** indicate statistical significance at the 10%, 5%, and 1% significance level, respectively.

#### Propensity Score Matching

To further eliminate the effects of systematic biases and confounding variables, we select a new sample group using propensity score matching (PSM). The treatment groups consist of the firms with famine CEOs and the control groups consists of the firms without famine CEOs. According to Table 12, columns (1) and (2) in present the regression results using the sample selected by *RET* and *C_SCORE* as the covariant. These two variables are more significant than others in the basic regression. Columns (3) and (4) report the regression results using the sample selected by firm registry, CEOs’ gender and education attainment as the covariant. These variables control the other firm and CEO characteristics. The coefficients of *Cohort* 3 are still positive and significant. After PSM, the regression results confirm our hypothesis.

**TABLE 12:** Robustness check: Propensity score matching.

	(1)	(2)	(3)	(4)
	*NCSKEW*_*t*+1_	*DUVOL*_*t*+1_	*NCSKEW*_*t*+1_	*DUVOL*_*t*+1_
*Cohort* 3_*t*_	0.068*	0.060*	0.136***	0.123***
	(1.72)	(1.75)	(3.61)	(3.79)
*NCSKEW*_*t*_	0.019		0.020	
	(0.77)		(0.84)	
*DUVOL*_*t*_		0.013		−0.007
		(0.48)		(–0.29)
*SIZE*_*t*_	0.038*	0.046**	0.042**	0.049***
	(1.86)	(2.56)	(2.01)	(2.75)
*LEV*_*t*_	−0.030	−0.036	0.092	0.052
	(–0.29)	(–0.40)	(0.91)	(0.60)
*ROE*_*t*_	−0.030	−0.084	0.035	−0.035
	(–0.22)	(–0.71)	(0.26)	(–0.31)
*MB*_*t*_	0.027**	0.021*	0.034***	0.024**
	(2.03)	(1.84)	(2.71)	(2.23)
*OTUM*_*t*_	−0.089	−0.070	−0.140**	−0.124**
	(–1.33)	(–1.18)	(–2.17)	(–2.24)
*RET*_*t*_	4.230***	3.592***	4.401***	3.608***
	(4.55)	(4.17)	(4.91)	(4.44)
*SIGMA*_*t*_	−0.913*	−0.582	−0.691	−0.345
	(–1.75)	(–1.27)	(–1.33)	(–0.77)
*ABACC*_*t*_	0.325	0.306	0.213	0.436*
	(1.07)	(1.15)	(0.76)	(1.79)
*C_SCORE_t_*	−4.673***	−4.873***	−4.453***	−4.609***
	(–6.33)	(–7.54)	(–6.00)	(–7.20)
*SOE*_*t*_	0.024	0.000	0.008	−0.020
	(0.54)	(0.00)	(0.19)	(–0.55)
*Registry*_*t*_	−0.024**	−0.022**	−0.031**	−0.030***
	(–2.04)	(–2.12)	(–2.52)	(–2.82)
*GENDER*_*t*_	−0.037	−0.047	−0.083	−0.104
	(–0.44)	(–0.65)	(–1.05)	(–1.52)
*EDU*_*t*_	0.010	0.013	−0.068	−0.044
	(0.25)	(0.35)	(–1.59)	(–1.18)
*Constant*	−4.215***	−4.445***	−4.458***	−4.632***
	(–5.89)	(–7.09)	(-6.07)	(-7.31)
Year	YES	YES	YES	YES
Industry	YES	YES	YES	YES
N	2190	2190	2190	2190
Adj R^2^	0.064	0.059	0.071	0.072

T-values are reported in parentheses. *, **, and *** indicate statistical significance at the 10%, 5%, and 1% significance level, respectively.

#### Alternative Proxy

Following Hutton et al. (2009), we use CRASH to measure the crash risk of stock price, which is set equal to 1 if the firm experiences one or more firm-specific weekly returns falling 3.09 standard deviations below the mean weekly firm-specific return for that fiscal year; otherwise, 0. According to the probit regression result in Table 13, we still find a positive and significant coefficient on Cohort 3.

**TABLE 13:** Robustness tests: Alternative proxy for stock price crash risk.

	*CRASH*
*Cohort 2_t_*	0.013
	−0.31
*Cohort 3_t_*	0.112**
	−2.41
*Cohort 4_t_*	0.206
	−0.61
*Controls*_*t*_	YES
Year	YES
Industry	YES
*N*	12393
Pseudo R^2^	0.206

** indicate statistical significance at the 5% significance level.

We have proven the mediating effect of bad news hoarding behavior in Section “Mediating Effect of Bad News Hoarding Behavior.” To verify its reliability, we use *information assessment* as a new proxy. The Shenzhen Stock Exchange publishes annual results of listed firms’ information disclosure assessment. The final assessment results are divided into four grades: excellent, good, qualified, and unqualified. In this robustness test, A dummy variable, *information assessment*, is equal to 1 when the information disclosure quality assessment of the listed companies are qualified or unqualified and 0 otherwise.

Table 14 presents the intermediary effects of regression testing on whether CEOs who experienced the Great Famine during their early-life increase firm-specific crash risk by hoarding bad news. In columns (1) and (4), the coefficient of *Cohort 3* is 0.105 (0.090) and is significant at the 5% level. In columns (2) and (5), the coefficient of *Cohort* 3 is also significantly positive, indicating that famine CEOs tend to hide bad news. When we add the intermediary factor into models (1) and (4), the coefficient of *Cohort* 3 decreases from 0.105 (0.090) to 0.100 (0.087). The Sobel *z*-value is 2.420 (2.048), which is significant at the 5% level. This finding suggests a partial mediation effect of hoarding bad news, which is consistent with our previous findings.

**TABLE 14:** Robustness check: Channel test.

	(1)	(2)	(3)	(4)	(5)	(6)
	Path a	Path b	Path c	Path a	Path b	Path c
	*NCSKEW*_*t*+1_	*information assessment*	*NCSKEW*_*t*+1_	*DUVOL*_*t*+1_	*information assessment*	*DUVOL*_*t*+1_
*Cohort 3*_*t*_	0.105**	0.140*	0.100**	0.090**	0.140*	0.087**
	(2.19)	(1.84)	(2.09)	(2.12)	(1.84)	(2.05)
*Information*_*t*_			0.111***			0.066**
			(3.35)			(2.26)
*Controls*_*t*_	YES	YES	YES	YES	YES	YES
Year	YES	YES	YES	YES	YES	YES
Industry	YES	YES	YES	YES	YES	YES
N	6527	6469	6527	6527	6469	6527
Adj R^2^	0.048	0.147	0.050	0.049	0.147	0.050
SobelZ			2.420**			2.048**

T-values are reported in parentheses. *, **, and *** indicate statistical significance at the 10%, 5%, and 1% significance level, respectively.

### Further Analysis

We have proved that the CEOs’ Great Famine experiences during early-life have significant effect on stock price crash risk by hoarding bad news. However, the degree of this influence is restricted by many factors. Upper echelons theory indicates that managers’ characteristics can represent their cognitive model, however, cognitive model is a variable with strong situational dependence. In different decision-making situations, managers with similar characteristics will appear bigger difference. The freedom of management is one of the important situational factors (Hambrick and Mason, 1984). Organizational research theory suggests that the CEO is more powerful, his control over the firm is stronger, his role and influence in the decision-making process are larger, and his judgment errors on decision outcomes should be greater (Sah and Stiglitz, 1991; Adams et al., 2005). Consistent with these theories, we concerned about the power of CEO. We hypothesize that when CEOs have more decision-making power, his or her early-life traumatic experiences during the Great Famine can have more significant effect on stock price crash risk. According to prior studies, CEO-Chair duality, founder status, the level of internal control and the proportion of the largest shareholder are effective measures of CEO power. Grouped regression is used to analyze how CEO power affect the relationships between early-life traumatic experience and firm’s stock price crash risk. Panel A, Panel B and Panel C in Table 15 show that CEO-chair duality, founder status and inefficient internal controls increase the effect of CEOs’ early-life famine experience on firm’s stock price crash risk, respectively. We find that the coefficients of *Cohort 3* have significant differences between the two groups with different levels of CEO power. When CEOs wields more decision-making power, his or her famine experiences during early-life have more significant effect on stock price crash risk. Power enhances the adverse effect of CEOs’ early-life traumatic experiences on crash risk.

**TABLE 15:** Moderating effect of CEO power.

Panel A CEO chair duality				
	**(1)**	**(2)**	**(3)**	**(4)**
	***NCSKEW*_*t*+1_**	***NCSKEW*_*t*+1_**	***DUVOL*_*t*+1_**	***DUVOL*_*t*+1_**
	***CEO-chair duality***	***independent***	***CEO-chair duality***	***independent***

*Cohort 3_t_*	0.143***	−0.005	0.118***	−0.002
	(3.33)	(−0.12)	(3.11)	(−0.04)
*Controls*_t_	YES	YES	YES	YES
Year	YES	YES	YES	YES
Industry	YES	YES	YES	YES
N	4863	9024	4863	9024
Adj R^2^	0.065	0.061	0.060	0.061
Difference	7.61***		6.47***	

**Panel B Founder CEO**				

	**(1)**	**(2)**	**(3)**	**(4)**
	***NCSKEW*_*t*+1_**	***NCSKEW*_*t*+1_**	***DUVOL*_*t*+1_**	***DUVOL*_*t*+1_**
	***founder CEOs***	***others***	***founder CEOs***	***others***

*Cohort 3_t_*	0.205***	0.039	0.167***	0.031
	(3.41)	(1.14)	(3.12)	(1.02)
*Controls*_t_	YES	YES	YES	YES
Year	YES	YES	YES	YES
Industry	YES	YES	YES	YES
N	4410	9477	4410	9477
Adj R^2^	0.037	0.078	0.040	0.075
Difference	6.45**		5.56**	

**Panel C Effective internal controls**				

	**(1)**	**(2)**	**(3)**	**(4)**
	***NCSKEW*_*t*+1_**	***NCSKEW*_*t*+1_**	***DUVOL*_*t*+1_**	***DUVOL*_*t*+1_**
	***effective controls***	***inefficient***	***effective controls***	***inefficient***

*Cohort 3_t_*	0.015	0.178***	0.013	0.148***
	(0.39)	(3.80)	(0.39)	(3.55)
*Controls*_t_	YES	YES	YES	YES
Year	YES	YES	YES	YES
Industry	YES	YES	YES	YES
N	6932	6955	6932	6955
Adj R^2^	0.069	0.062	0.071	0.058
Difference	8.97**		7.95***	

**Panel D Proportion of the largest shareholder**				

	**(1)**	**(2)**	**(3)**	**(4)**
	***NCSKEW*_*t*+1_**	***NCSKEW*_*t*+1_**	***DUVOL*_*t*+1_**	***DUVOL*_*t*+1_**
	***higher proportion***	***lower proportion***	***higher proportion***	***lower proportion***

*Cohort 3_t_*	0.093**	0.084*	0.074**	0.066*
	(2.23)	(1.95)	(2.03)	(1.72)
*Controls*_t_	YES	YES	YES	YES
Year	YES	YES	YES	YES
Industry	YES	YES	YES	YES
N	6744	7143	6744	7143
Adj R^2^	0.061	0.061	0.059	0.058
Difference	0.03		0.03	

T-values are reported in parentheses. *, **, and *** indicate statistical significance at the 10%, 5%, and 1% significance level, respectively.

However, the Panel D in Table 15 cannot prove this conclusion. In response to this unanticipated result and considered our unique system background, we infer that may be due to the fact that the role of the first largest shareholder differs between state-owned enterprises and private firms, with the different roles causing the effects to cancel each other out. We further divide the sample according to the nature of ownership. In state-owned enterprises, the first largest shareholder plays a “supervisory effect,” i.e., the higher the shareholding of the first largest shareholder, the less the CEO power; while the establishment and success in business of the private enterprises in China rely heavily on the personal charisma of the founder, and generally have the characteristic of one share dominated by the entrepreneur (or the family). The first largest shareholder and the CEO usually represent the same interest subject. Therefore, in private enterprises, the higher the shareholding of the first largest shareholder, the more the CEO power. Then grouped regressions are used to test the moderating effect of CEO power. Table 16 presents the regression results. We also find that the coefficients of *Cohort 3* have significant differences between the two groups with different levels of CEO power. When CEOs wields more decision-making power, his or her famine experiences during early-life have more significant effect on stock price crash risk.

**TABLE 16:** The moderating effect of managers’ power measured by the first shareholder’s shareholding proportion.

	state-owned enterprises				private enterprises			
	(1)	(2)	(3)	(4)	(5)	(6)	(7)	(8)
	*NCSKEW*_*t*+1_	*NCSKEW*_*t*+1_	*DUVOL*_*t*+1_	*DUVOL*_*t*+1_	*NCSKEW*_*t*+1_	*NCSKEW*_*t*+1_	*DUVOL*_*t*+1_	*DUVOL*_*t*+1_
	Lower proportion	Higher proportion	Lower proportion	higher proportion	Lower proportion	Higher proportion	Lower proportion	Higher proportion
*Cohort*_3,*t*_	0.123**	0.003	0.095*	0.000	0.01	0.222***	0.018	0.176**
	(2.21)	(0.05)	(1.96)	(−0.00)	(0.13)	(2.81)	(0.27)	(2.48)
*Controls*_t_	YES	YES	YES	YES	YES	YES	YES	YES
Year	YES	YES	YES	YES	YES	YES	YES	YES
Industry	YES	YES	YES	YES	YES	YES	YES	YES
*N*	3585	4380	3585	4380	2792	2467	2792	2467
*Adj R^2^*	0.082	0.082	0.075	0.081	0.045	0.049	0.053	0.046
Difference	3.19*		2.61*		4.87**		3.45*	

T-values are reported in parentheses. *, **, and *** indicate statistical significance at the 10%, 5%, and 1% significance level, respectively.

## Conclusion

Sixty years have passed since the end of the Great Famine. However, the traumatic of this disaster on those who experienced it during early-life is profound and lasting. Drawing on the idea of natural experiments, the paper takes this Great Famine as an external traumatic event which cannot be selected or controlled by human and test the impact of CEOs’ famine experiences during early-life on firm-specific stock price crash risk, relying on a large sample of CEOs from A-share listed companies. The study found that CEOs who had experienced the Great Famine during their early-life tended to hide bad news, increasing the stock price crash risk. The higher the severity of their famine experience, the higher the crash risk; the longer duration of their famine experience, the higher the crash risk. When CEOs wields more decision-making power, the effect of the traumatic experiences will be more significant. From the perspective of CEOs’ early-life traumatic experience, we reveal the mechanism of post-traumatic compensation psychology and irrational defensive psychology causing CEOs to hide bad news. This new explanation for the stock price crash risk extends the bad news hoarding conjecture. Our findings are important for the understanding of how early-life traumatic experiences affect a CEO’s decision-making processes and add to the evidence of the economic consequences of early-life traumatic experiences. Furthermore, our study is helpful for regulators to explore the deep causes of stock price collapse at the level of managers and provide some reference for effectively reducing the stock price crash risk. Meanwhile, it provides reference for improving corporate governance and optimizing management appointment.

## Data Availability Statement

The original contributions presented in the study are included in the article/supplementary material, further inquiries can be directed to the corresponding author/s.

## Author Contributions

GH contributed to the conception of the study. FC performed the data analyses and wrote the manuscript. WR contributed significantly to analysis and manuscript preparation. LZ helped perform the analysis with constructive discussions. All authors contributed to the article and approved the submitted version.

## Conflict of Interest

The authors declare that the research was conducted in the absence of any commercial or financial relationships that could be construed as a potential conflict of interest.

## Publisher’s Note

All claims expressed in this article are solely those of the authors and do not necessarily represent those of their affiliated organizations, or those of the publisher, the editors and the reviewers. Any product that may be evaluated in this article, or claim that may be made by its manufacturer, is not guaranteed or endorsed by the publisher.

## Appendix

**Appendix A:**

Variable	Definition
*NCSKEW*	Negative coefficient of skewness of firm-specific weekly returns in the fiscal year. See Eq. (2) for details.
*DUVOL*	Down-to-up volatility measure of the crash likelihood over the fiscal year. See Eq. (3) for details.
*Cohort*	Birth cohort in Table 1.
*Famine_severity*	excess death rate, the average death rate of the three-year famine in the province (1959–1961) minus the average death rate of the five-year pre-famine period (1954–1958)
*Famine_duration*	The provinces with famine lasting for more than three years are assigned a value of 1, whereas those with famine lasting three years or less are assigned a value of 0.
*SIZE*	Natural logarithm of the book value of total assets.
*LEV*	Firm financial leverage.
*ROE*	Return on equity.
*MB*	Market-to-book ratio.
*OTUM*	Detrended stock trading volume, calculated as the average monthly share turnover for the current fiscal year minus the average monthly share turnover for the previous fiscal year.
*SIGMA*	Standard deviation of firm-specific weekly returns.
*RET*	Mean of firm-specific weekly returns.
*ABACC*	Absolute value of discretionary accruals, where discretionary accruals are estimated from the modified Jones model.
*C_SCORE*	Conditional accounting conservatism score.
*SOE*	Firm ownership that is equal to 1 if the firm is an SOE and 0 otherwise.
*Registry*	Marketization index of China’s province.
*GENDER*	Gender of the CEO, where equals to 1 for male and 0 otherwise.
*EDU*	CEO’s educational attainment, where equals to 1 for master or doctoral degree and 0 otherwise.
